# Effects of *Allium mongolicum* Regel ethanol extract on three flavor-related rumen branched-chain fatty acids, rumen fermentation and rumen bacteria in lambs

**DOI:** 10.3389/fmicb.2022.978057

**Published:** 2022-09-15

**Authors:** Yabo Zhao, Yanmei Zhang, Erdene Khas, Changjin Ao, Chen Bai

**Affiliations:** Key Laboratory of Animal Feed and Nutrition of Inner Mongolia Autonomous Region, College of Animal Science, Inner Mongolia Agricultural University, Hohhot, China

**Keywords:** *Allium mongolicum* Regel ethanol extract, branched-chain fatty acids, mutton flavor, rumen bacteria, lamb

## Abstract

The objective of this study was to evaluate the effect of *Allium mongolicum* Regel ethanol extract (AME) on the concentration of three branched-chain fatty acids (BCFAs) related to flavor, fermentation parameters and the bacteria and their correlations in the rumen of lambs. A total of thirty 3-month-old male, Small-tailed Han sheep (33.60 ± 1.23 kg) were randomly distributed into 2 groups as follows: control group (CON) was fed a basal diet and AME group was fed a basal diet supplemented with 2.8 g⋅lamb^–1^⋅d^–1^ *A. mongolicum* Regel ethanol extract. AME supplementation decreased (*P* = 0.022) 4-methyloctanoic acid (MOA) content and tended to lower (*P* = 0.055) 4-methylnonanoic acid (MNA) content in the rumen. Compared to CON group, the ruminal concentrations of valerate and isovalerate were higher (*P* = 0.046 and *P* = 0.024, respectively), and propionate was lower (*P* = 0.020) in the AME group. At the phylum level, the AME group had a lower abundance of *Bacteroidetes* (*P* = 0.014) and a higher abundance of *Firmicutes* (*P* = 0.020) than the CON group. At the genus level, the relative abundances of *Prevotella* (*P* = 0.001), *Christensenellaceae_R-7_group* (*P* = 0.003), *Succiniclasticum* (*P* = 0.004), and *Selenomonas* (*P* = 0.001) were significantly lower in the AME group than in the CON group, while the relative abundances of *Ruminococcus* (*P* < 0.001), *Quinella* (*P* = 0.013), and *Lachnospiraceae_XPB1014_group* (*P* = 0.001) were significantly higher. The relative abundances of *Prevotella* (*P* = 0.029, *R* = 0.685; *P* = 0.009, *R* = 0.770), *Christensenellaceae_R-7_group* (*P* = 0.019, *R* = 0.721; *P* = 0.029, *R* = 0.685), and *Succiniclasticum* (*P* = 0.002, *R* = 0.842; *P* = 0.001, *R* = 0.879) was positively correlated with MOA and MNA levels, and the relative abundance of *Lachnospiraceae_XPB1014_group* (*P* = 0.033, *R* = −0.673) was negatively correlated with MOA. The relative abundance of *Christensenellaceae_R-7_group* (*P* = 0.014, *R* = −0.744) and *Prevotellaceae_UCG-003* (*P* = 0.023, *R* = −0.706) correlated negatively with the EOA content. In conclusion, these findings suggest that the AME affected the concentration of BCFAs, fermentation parameters and the rumen bacteria in the rumen of lambs.

## Introduction

Sheep meat and milk have a characteristic “mutton flavor,” which is mainly associated with branched-chain fatty acids (BCFAs), especially 4-methyloctanoic acid (MOA), 4-ethyloctanoic acid (EOA) and 4-methylnonanoic acid (MNA) (Salles et al., 2002). MOA and MNA are *de novo* synthesized by the use of methylmalonyl-CoA in chain lengthening with acetyl-CoA using propionate as a precursor in the liver (Massart et al., 1983; Vlaeminck et al., 2006). Propionate from rumen fermentation through the rumen epithelium into the portal vein arrives at the liver, and then the liver synthesize BCFAs when propionate concentrations exceed the capacity of gluconeogenesis (Wong et al., 1975; Aschenbach et al., 2010). The biosynthesis pathway of EOA is less well known and may follow the same biosynthesis pathway of MOA and MNA, which uses butyrate and ethylmalonyl-CoA instead of propionate and methylmalonyl-CoA (Kaffarnik et al., 2015). Methylmalonyl-CoA and ethylmalonyl-CoA originate from the carboxylation of propionyl-CoA and butyryl-CoA, respectively, by acetyl-CoA carboxylase (Dewulf et al., 2019). In addition, ruminal microbiota also participate in the formation of BCFAs, which by using the carbon skeleton of iso-butyrate, iso-valerate and branched-chain amino acids (Shivani et al., 2016).

The composition of a diet is one of the most important factors affecting the concentration of BCFAs. High-concentrate diets lead to a higher proportion of VFAs, especially propionate, in the rumen than low-concentrate diets (Plaizier et al., 2008), which will provide sufficient precursors for the synthesis of BCFAs. Young et al. (2003) reported that ram lambs fed with an alfalfa pellet or a corn-based diet had greater concentrations of MOA and MNA in the subcutaneous fat than ram lambs grazing ryegrass/clover pasture. Teng et al. (2020) found that milk from lambs offered perennial ryegrasses/white clover has lower levels of MOA and MNA than milk from lambs offered Lucerne silage and soy meal. In recent years, researchers have made substantial efforts to reduce mutton flavor by adding natural plant extracts, such as tannins, flavonoids, and essential oils, to lamb diets. Vasta et al. (2013) stated that essential oils from *Rosmarinus officinalis* or *Artemisia herba alba* can change the volatile compound composition in lamb meat. Del Bianco et al. (2021) reported that supplementation with 4% tannins from *Acacia mearnsii*, *Castanea sativa*, or *Caesalpinia spinosa* in the diet decreased the concentrations of MOA and 8-methylnonanoic acid, which also conferred a “mutton” flavor (Brennand et al., 1989) to the perirenal fat of Sarda × Comisana lambs.

*Allium Mongolicum* Regel is a traditional Mongolian medicinal herb belonging to the genus *Allium* of the *Liliaceae* family and grows extensively in northwest China (Wang et al., 2019). *A. Mongolicum* Regel contains polysaccharides, flavonoids, polyphenols, and other bioactive compounds (Li et al., 2019). Our previous studies indicated that *A. Mongolicum* Regel ethanol extract (AME) significantly decreased the concentration of BCFAs in the *longissimus dorsi* muscle and *dorsal subcutaneous, omental* and *perirenal* adipose tissues of Small-tailed Han sheep (Liu et al., 2019; Liu and Ao, 2021). However, it is not clear whether AME affects the synthesis of three BCFAs related to flavor in the rumen of lambs. Thus, the objective of this study was to investigate the effects of AME on the concentrations of three rumen BCFAs related to flavor, rumen fermentation parameters, the diversity of rumen bacteria and their correlations in lambs.

## Materials and methods

All experimental procedures involving animals were evaluated and approved according to the guidelines of the Animal Care and Use Committee of Inner Mongolia Agriculture University (Hohhot, China). This study was conducted at a commercial farm in Bayannaoer, Inner Mongolia Autonomous Region, China (latitude 40°13′–42°28′; longitude 105°12′–109°53′) between April and June 2021.

### Animals and experimental design

A total of thirty 3-month-old male, Small-tailed Han sheep (33.60 ± 1.23 kg) were randomly distributed into 2 groups as follows: control group (CON) was fed a basal diet and AME group was fed a basal diet supplemented with 2.8 g⋅lamb^–1^⋅d^–1^ *A. Mongolicum* Regel ethanol extract. One group was composed of three pens, with five lambs in each pen (3.95 m × 5.12 m). *A. Mongolicum* Regel powder was purchased from Hao Hai Biological Company (Alxa League, Inner Mongolia, China), and the ethanol extraction process was conducted as previously described by Ding et al. (2021). The obtained AME primarily contained 26.43% flavonoids, 18.57% organic acids and their derivatives, 14.43% nucleotides and their derivatives, 11.14% amino acids and others. The dose of the ethanol extract (2.8 g⋅lamb^–1^⋅d^–1^) in the diet has been proven to be most beneficial for the lamb based on our previous studies (Zhao et al., 2021). We mixed 2.8 g AME with 50 g concentrate for each lamb. Thus, the total dose of five lambs (a pen) is 14 g AME mixed with 250 g concentrate, divided into two equal amounts, and provided for each pen twice daily to ensure that the lambs completely consumed the AME. Each pen of CON group was fed 250 g of concentrate without AME in the same manner. Then, total mixed ration was provided, and the lambs were allowed to feed *ad libitum*. The basal diet met the requirements for sheep, as described by the National Research Council (NRC, 2007), and its composition and nutritional level are shown in Table 1.

**TABLE 1:** Composition and nutrient levels of the basal diet (dry matter basis %).

Item	Content (%)
Chinese wildrye	25.00
Caragana	17.80
Whole corn silage	23.60
Wheat bran	5.15
Sunflower seed meal	21.35
Pea stem and leaf	2.64
Red jujube	2.04
CaHPO_4_	0.74
NaCl	0.68
Premix^a^	1.0
Total	100.00
Nutrient level	
DE^b^ (MJ/kg)	13.46
CP	16.87
NDF	38.72
ADF	27.51
Ca	1.33
P	0.53

^*a*^Nutritional composition of premix per kilogram: Mn 30.00 mg, Fe 25.00 mg, Zn 29.00 mg, Cu 8.00 mg, Co 0.10 mg, I 0.04 mg, VA 3200 IU, VD 1200 IU, VE 20 IU.

^*b*^DE was a calculated value, and the others were measured values; DE, digestible energy.

The experimental period lasted for 75 days, with 15 days of adaptation to the experimental environment and 60 days for the experimental feeding period. The lambs were given free access to water and TMR was provided twice daily at 7:00 and 18:00. During the experimental period, feed was provided to the lambs and refusals were recorded to calculate the dry matter intake (DMI). The body weight of each lamb was also recorded before morning feeding at 0, 15, 30, 45, and 60 days, to calculate average daily gain (ADG), meanwhile, initial body weight (IBW), and final body weight (FBW) were recorded.

### Sampling

At 60 days of the experimental period, rumen fluid samples were collected before the morning feeding using an oral stomach tube connected to a 50 mL syringe from six randomly selected lambs from each group. The initial part of the rumen fluid was discarded to avoid contamination with saliva, and then rumen fluid (approximately 50 mL) was collected and filtered through four layers of gauze. The pH was measured using a portable pH meter (PHB-4; INESA Scientific Instrument Co., Ltd, Shanghai, China). 10 mL of rumen fluid was mixed with 2 mL of 25% HPO_3_ and stored at −20°C until volatile fatty acid (VFA) analysis. 10 mL of rumen fluid was stored at −20°C until ammonium nitrogen (NH_3_-N) analysis. The remaining rumen fluid was immediately frozen in liquid nitrogen and stored at −80°C until BCFAs analysis and microbial community analysis.

### Chemical analyses of feeds

Samples of feed were dried at 55°C for 48 h and then ground to pass through a 2 mm sieve before chemical analysis. Crude protein (N × 6.25, method 981.10) and minerals content were measured using an analytical method provided by the Association of Official Agricultural Chemists (AOAC, 2000). The neutral detergent fiber, acid detergent fiber were determined as described by Van Soest et al. (1991).

### Branched-chain fatty acid analyses

Three BCFAs, namely, MOA, MNA, and EOA, in the rumen were analyzed by gas chromatography–mass spectrometry (GC–MS). Prior to GC–MS analysis, fatty acids in the rumen were converted to their corresponding methyl esters, which was carried out according to the method of Kaffarnik et al. (2014). Undecanoic acid (Sigma–Aldrich, USA) was used as an internal standard.

A GC–MS system (Thermo TRANCE GC 1300) equipped with an autosampler was used in EI mode (70 eV). Solutions (1 μL) were injected in split mode (split ratio 10:1) onto a TG-WAX column (30 m, 0.25 mm i.d., 0.25 μm film thickness). The GC oven was held at 60°C for 2 min, and then, the temperature was raised at 20°C min^–1^ to 250°C and held for 5 min. The flow rate of the helium carrier gas was 1.2 mL/min. The mass spectrometer transfer line was operated at 250°C. Mass spectra were acquired using an ion source temperature of 220°C. GC–MS analyses were performed in full-scan mode (m/z 50–350).

### Ruminal fermentation analyses

Ruminal NH_3_-N concentrations were analyzed using a microplate reader (Epoch, BioTek Instruments, Inc., USA) according to the method described by Chaney and Marbach (1962). The concentration of VFA was determined using a gas chromatograph (GC-2014; Shimadzu, Japan) with a fused silica column (60 m × 0.25 mm × 0.50 μm; DB-FFAP, Agilent Technologies, USA) according to Erwin et al. (1961). 2-Ethyl butyric acid was used as an internal standard. The column temperature was increased from 80 to 180°C at 20°C/min and held for 3 min. The injector and detector temperatures were set at 220 and 250°C, respectively.

### 16S rDNA extraction and sequencing

Microbial community genomic DNA was extracted from rumen fluid samples using the E.Z.N.A.^®^ soil DNA Kit (Omega Bio-Tek, Norcross, GA, U.S.) according to the manufacturer’s instructions. The DNA quality and quantity were determined by a NanoDrop 2000 UV–vis spectrophotometer (Thermo Fisher Scientific, Wilmington, USA). The V3–V4 region of the bacterial 16S rRNA gene was amplified with the primer pair 338F (5′-ACTCCTACGGGAGGCAGCAG-3′) and 806R (5′-GGACTACHVGGGTWTCTAAT-3′) by an ABI GeneAmp^®^ 9700 PCR thermocycler (ABI, CA, USA). The PCR amplification of the 16S rRNA gene was performed as follows: initial denaturation at 95°C for 3 min, followed by 27 cycles of denaturing at 95°C for 30 s, annealing at 55°C for 30 s and extension at 72°C for 45 s, and a single extension at 72°C for 10 min, ending at 4°C. The PCR mixtures contained 4 μL of 5 × *TransStart* FastPfu buffer, 2 μL of 2.5 mM deoxynucleoside triphosphates, 0.8 μL of each primer (5 μM), 0.4 μL of *TransStart* FastPfu DNA polymerase, 10 ng of template DNA, and ddH_2_O to reach a total volume of 20 μL. The PCR products were purified using the AxyPrep DNA Gel Extraction Kit (Axygen Biosciences, Union City, CA, USA) and quantified using a Quantus™ Fluorometer (Promega, USA). Purified amplicons were sequenced on an Illumina MiSeq PE300 platform (Illumina, San Diego, USA).

### Sequencing data analyses

Fastp v0.19.6 software^1^ and FLASH v1.2.11 software^2^ were used for quality control and splicing, respectively. UPARSE v7.0.1090 software was used to cluster operational taxonomic units (OTUs) with 97% similarity (Edgar, 2013). The taxonomy of each OTU representative sequence was analyzed by ribosomal database project (RDP) classifier v2.11^3^ compared with Silva v138^4^ using a comparison threshold of 70% (Han et al., 2015). Alpha diversity analysis was performed by Mothur v1.30.2^5^.

### Statistical analysis

Sequencing data followed normality (Shapiro–Wilk’s tests, *P* > 0.2) after log transformations, and their differences were homogenous (Levene’s test, *P* > 0.05). Non-transformed values are shown in this study. Data for growth performance, the concentration of BCFAs, rumen fermentation parameters and bacterial communities between CON and AME group lambs were analyzed using one-way ANOVA by SPSS 26.0 (IBM, New York, USA). The statistical model was Y_i_ = μ + A_i_ + e, where Y_i_ is the dependent variable; μ is the overall mean; A_i_ is the fixed effect of treatment (*i* = 1.2, CON or AME); and e is the random effect. The degrees of freedom of ANOVA are as follows: total: df_T_ = n - 1 (*n* = total sample size); group: df_G_ = k - 1 (*k* = total group size); error: df_E_ = df_T_ - df_G_. Statistical significance was defined at *P* < 0.05, and trending toward significance was defined at 0.05 ≤ *P* ≤ 0.1. Spearman’s rank correlation coefficient analysis between BCFA levels, VFA levels and rumen bacterial taxa abundances was carried out using tools on the Majorbio platform (Majorbio Bio-Pharm Technology Co., Ltd.; Shanghai, China)^6^. Significant correlations were defined at *P* < 0.05.

## Results

### Growth performance

The results of the effect of AME on the growth performance of lambs are presented in Table 2. There were no differences in FBM and DMI between the CON and AME groups. Compared with the CON group, AME supplementation tended to increase (*P* = 0.098) the ADG of lambs.

**TABLE 2:** Effect of AME on the growth performance of lambs.

Items	CON	AME	SEM	*P*-value
IBW^a^, kg	33.40	34.14	0.37	0.342
FBW^b^, kg	50.22	51.28	1.14	0.526
ADG^c^, g	240.6	287.2	0.01	0.098
DMI^d^, g/d	1095.23	1117.38	21.42	0.659

CON, basal diet; AME, basal diet + 2.8 g⋅lamb^–1^⋅d^–1^ AME.

^a^IBW, initial body weight.

^b^FBW, final body weight.

^c^ADG, average daily gain.

^d^DMI, dry matter intake.

### Three branched-chain fatty acids concentrations and fermentation parameters in the rumen

**FIGURE 1:**
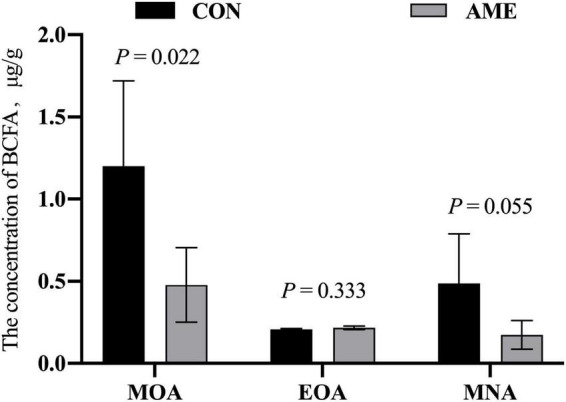
Effect of AME on the concentration of BCFAs in the rumen of lambs. CON, basal diet; AME, basal diet + 2.8 g⋅lamb^–1^⋅d^–1^ *A. mongolicum* Regel ethanol extract (AME).

There was no difference in the concentration of EOA in rumens between the CON and AME groups (Figure 1). AME supplementation decreased (*P* = 0.022) the concentration of MOA and tended to lower (*P* = 0.055) the concentration of MNA in the rumen.

The results of the rumen fermentation parameters are presented in Table 3. There were no differences in ruminal pH, TVFA level, acetate level, butyrate level, iso-butyrate level, or acetate/propionate ration between the CON and AME groups. The concentration of rumen NH_3_-N (*P* = 0.078) tended to be lower in the AME group than in the CON group. Lambs consuming the AME had higher ruminal concentrations of valerate and iso-valerate (*P* = 0.046 and *P* = 0.024, respectively) than lambs consuming the CON diet and had a lower ruminal concentration of propionate (*P* = 0.020).

**TABLE 3:** Effect of AME on rumen fermentation in lambs.

Items	CON	AME	SEM	*P*-value
pH	7.00	7.17	0.07	0.276
NH_3_-N^a^, mg/dL	7.12	6.51	0.17	0.078
TVFA^b^, mmol/L	61.60	60.42	0.81	0.424
Acetate, mmol/L	37.94	37.03	0.54	0.494
Propionate, mmol/L	12.07	10.84	0.29	0.020
Butyrate, mmol/L	8.13	8.58	0.36	0.565
Iso-butyrate, mmol/L	1.27	1.35	0.05	0.376
Valerate, mmol/L	0.67	0.77	0.03	0.046
Iso-valerate, mmol/L	1.52	1.84	0.08	0.024
Acetate/Propionate	3.16	3.42	0.09	0.150

CON, basal diet; AME, basal diet + 2.8 g⋅lamb^–1^⋅d^–1^ AME.

^a^NH_3_-N, ammonia nitrogen.

^b^TVFA, total volatile fatty acid.

### Abundance and diversity of rumen bacteria

In total, 664,062 raw sequence reads were obtained by sequencing from the lamb ruminal fluid in the CON and AME groups. After quality control and filtering, a total of 556,763 clean tags were generated, and an average of 46,397 clean tags were analyzed for each sample. According to 97% similarity, 1,243 OTUs were identified in the two groups (Table 4). No significant differences were found in the OTU number, ACE, Chao 1 index, or Shannon and Simpson diversity indices between the two treatment groups. The coverage (*P* = 0.07) tended to higher in AME group than CON group.

**TABLE 4:** Effect of AME on OTUs and alpha indices in lambs.

Items	CON	AME	SEM	*P*-value
OTU^a^	1,108	1,150	4.62	0.358
ACE^b^	766.02	711.42	5.90	0.480
Coverage	99.70%	99.79	0.0002	0.070
Chao1	770.60	714.82	7.29	0.487
Shannon	4.30	4.38	0.11	0.766
Simpson	0.04	0.04	0.01	0.905

CON, basal diet; AME, basal diet + 2.8 g⋅lamb^–1^⋅d^–1^ AME.

^a^OTUs, operational taxonomic units.

^b^ACE, abundance-based coverage estimator.

At the phylum level, the relative abundances of the top 10 rumen bacterial phyla are presented in Table 5. *Bacteroidetes* and *Firmicutes* were the predominant phyla for the two groups and accounted for approximately 96% of the total bacterial abundance. The relative composition of the bacterial phyla was changed when the diet was supplemented with AME. Compared to that in the CON group, the richness of *Bacteroidetes* (*P* = 0.014) was significantly decreased in the AME group, while the richness of *Firmicutes* (*P* = 0.020) was significantly increased. The relative abundances of *Proteobacteria* (*P* = 0.001) and *Cyanobacteria* (*P* = 0.001) were higher in the AME group than in the CON group, and that of *Desulfobacterota* (*P* = 0.052) tended to be higher.

**TABLE 5:** Effect of AME on bacterial abundance at the phylum level in the rumen of lambs (%).

Items	CON	AME	SEM	*P*-value
Bacteroidetes	55.18	36.84	4.13	0.014
Firmicutes	42.35	59.12	3.92	0.020
Actinobacteriota	0.66	0.56	0.18	0.801
Desulfobacterota	0.56	0.92	0.10	0.052
Synergistota	0.36	0.51	0.14	0.623
Spirochaetota	0.31	0.25	0.05	0.578
Proteobacteria	0.18	0.72	0.12	0.001
Patescibacteria	0.17	0.34	0.07	0.266
unclassified_k__norank_d__Bacteria	0.07	0.07	0.01	0.860
Cyanobacteria	0.06	0.25	0.04	0.001

CON, basal diet; AME, basal diet + 2.8 g⋅lamb^–1^⋅d^–1^ AME.

At the genus level, 22 rumen bacterial genera with a relative abundance > 1% in at least one group are presented in Table 6. The dominant bacterial genera were *Prevotella*, *norank_f_bacteroidales_RF16_group*, *Rikenellaceae_RC9_gut_group* and *Ruminococcus* in both groups. The relative abundances of *Prevotella* (*P* = 0.001), *norank_f_bacteroidales_RF16_group* (*P* = 0.001), *Christensenellaceae_R-7_group* (*P* = 0.003), *norank_f_p-251-o5* (*P* = 0.006), *Succiniclasticum* (*P* = 0.004), and *Selenomonas* (*P* = 0.001) were significantly lower in the AME group than in the CON group. In addition, the relative abundances of *Ruminococcus* (*P* < 0.001), *Quinella* (*P* = 0.013), *norank_f_Muribaculaceae* (*P* < 0.001), *unclassified_f_Ruminococcus* (*P* < 0.001), and *Lachnospiraceae_XPB1014_group* (*P* = 0.001) were significantly higher in the AME group than in the CON group.

**TABLE 6:** Effect of AME on bacterial abundance at the genus level in the rumen for lambs (>1% at least in one group).

Items	CON	AME	SEM	*P*-value
Prevotella	22.47	11.99	2.02	0.001
norank_f__Bacteroidales_RF16_group	8.63	3.90	0.90	0.001
Rikenellaceae_RC9_gut_group	7.00	5.65	0.46	0.155
Ruminococcus	5.56	16.39	1.60	<0.001
Christensenellaceae_R-7_group	4.50	1.71	0.56	0.003
norank_f__p-251-o5	3.89	1.35	0.53	0.006
norank_f__F082	3.53	2.58	0.32	0.150
Prevotellaceae_UCG-003	3.46	2.57	0.31	0.167
Succiniclasticum	2.94	1.93	0.21	0.004
Quinella	2.77	9.55	1.51	0.013
Selenomonas	2.76	0.76	0.20	0.001
Veillonellaceae_UCG-001	2.65	1.82	0.25	0.102
Veillonella	2.65	1.82	0.31	0.199
unclassified_f__Selenomonadaceae	2.24	1.63	0.35	0.422
Eubacterium_Coprostanoligenes_group	2.21	2.02	0.22	0.701
NK4A214_group	2.21	2.11	0.36	0.901
Prevotellaceae_UCG-001	2.18	2.59	0.43	0.657
Lachnospiraceae_NK3A20_group	2.08	1.79	0.28	0.635
Anaerovibrio	1.47	2.00	0.40	0.541
norank_f__Muribaculaceae	1.39	3.52	0.38	<0.001
unclassified_f__Ruminococcaceae	1.38	4.24	0.53	<0.001
Lachnospiraceae_XPB1014_group	0.37	2.12	0.33	0.001

CON, basal diet; AME, basal diet + 2.8 g⋅lamb^–1^⋅d^–1^ AME.

### Relationships between volatile fatty acid levels, rumen bacteria and branched-chain fatty acid levels

**FIGURE 2:**
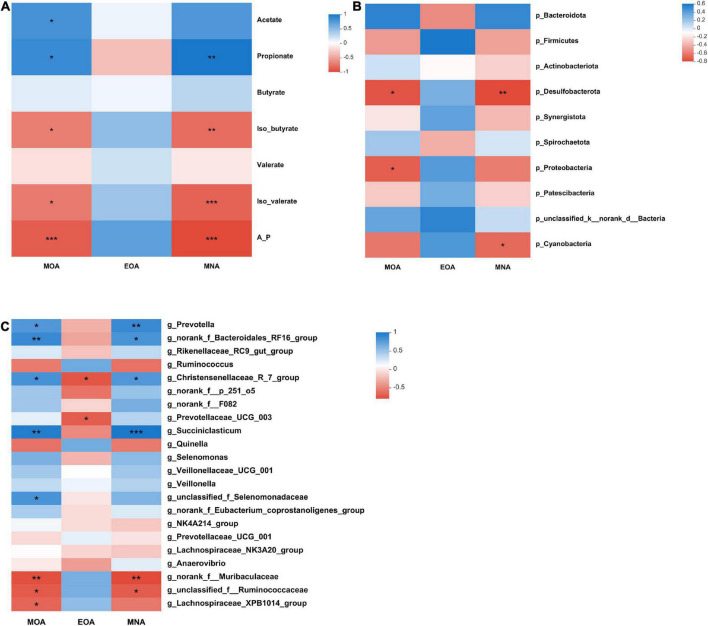
Correlations between VFA contents, microbial taxa and BCFA contents **(A–C)**. **(A)** Correlation between the concentration of BCFAs and VFAs. **(B)** Correlation between the concentration of BCFAs and the top 10 microbial taxa at the phylum level. **(C)** Correlation between the concentration of BCFAs and genera with relative abundances > 1% (at least in one group). Significant correlations are shown by *P < 0.05, **P < 0.01, and ***P < 0.001. Blue represents positive correlation coefficients. Red represents negative correlation coefficients.

The relationships between BCFA levels, rumen fermentation parameters and the bacterial community are presented in Figure 2. Iso-butyrate (*P* = 0.015, *R* = −0.736; *P* = 0.003, *R* = −0.827) and iso-valerate (*P* = 0.011, *R* = −0.758; *P* = 0.001, *R* = −0.879) levels correlated negatively with the MOA and MNA levels. At the phylum level, the relative abundances of *Desulfobacterota* (*P* = 0.016, *R* = −0.733) and *Proteobacteria* (*P* = 0.025, *R* = −0.697) correlated negatively with the MOA content. The relative abundances of *Desulfobacterota* (*P* = 0.009, *R* = −0.770) and *Cyanobacteria* (*P* = 0.038, *R* = −0.661) negatively correlated with the MNA content.

At the genus level, there were positive correlations among the following: MOA content with the *Prevotella* (*P* = 0.029, *R* = 0.685), *norank_f_Bacteroidales_RF16_group* (*P* = 0.009, *R* = 0.770), *Christensenellaceae_R-7_group* (*P* = 0.019, *R* = 0.721), *Succiniclasticum* (*P* = 0.002, *R* = 0.842), and *unclassified_f_Selenomonadaceae* (*P* = 0.022, *R* = 0.709) abundances and MNA content with the *Prevotella* (*P* = 0.009, *R* = 0.770), *norank_f_Bacteroidales_RF16_group* (*P* = 0.022, *R* = 0.709), *Christensenellaceae_R-7_group* (*P* = 0.029, *R* = 0.685), and *Succiniclasticum* (*P* = 0.001, *R* = 0.879) abundances. Moreover, there were negative correlations between the MOA content with the *norank_f__Muribaculaceae* (*P* = 0.009, *R* = −0.770), *unclassified_f__Ruminococcaceae* (*P* = 0.019, *R* = −0.721), and *Lachnospiraceae_XPB1014_group* (*P* = 0.033, *R* = −0.673) abundances; EOA content with the *Christensenellaceae_R-7_group* (*P* = 0.014, *R* = −0.744) and *Prevotellaceae_UCG-003* (*P* = 0.023, *R* = −0.706) abundances; and MNA content with the *norank_f__Muribaculaceae* (*P* = 0.006, *R* = −0.794) and *unclassified_f__Ruminococcaceae* (*P* = 0.022, *R* = −0.709) abundances.

## Discussion

### Effect of *Allium mongolicum* Regel ethanol extract on the growth performance of Lambs

In this study, there were no differences in the FBW and DMI between the CON and AME groups. In accordance with this study, Ding et al. (2021) reported that AME did not affect the FBW and DMI, which suggested that AME had no effect on the palatability of the diet. AME supplementation tended to increase the ADG of lambs, which may be attributed to the high content of flavonoids in the AME. Muqier et al. (2017) reported that flavonoids from *Allium Mongolicum* Regel promote the secretion of growth hormone, insulin-like growth factor and adrenocorticotropic hormone, leading to a significant increase in the ADG. This result proved that AME could improve the growth performance of lambs.

### Effect of *Allium mongolicum* Regel ethanol extract on three branched-chain fatty acids concentrations and fermentation parameters in the rumen of lambs

The rumen is a complex ecosystem that has a strong relationship with the formation of BCFAs. Acetate, propionate and butyrate are produced by rumen fermentation, which through the rumen epithelium into the portal vein arrives at the liver to increase the synthesis of BCFAs in the liver of lambs (Chilliard et al., 2003). Berthelot et al. (2001) further verified this view and suggested that a diet supplemented with propionate significantly increased the content of BCFAs in *subcutaneous* and visceral fat of lambs. This could also explain why concentrate-based diets promote animal growth, while meat has a strong mutton flavor. In the present study, we observed that AME supplementation decreased the concentrations of MOA and MNA in the rumen of lambs. The lower concentrations of MOA and MNA in the AME group might be due to the active ingredients, such as flavonoids and organic acids, of the AME inhibiting the isomerization of unsaturated fatty acids to BCFAs after triglyceride degradation.

In the present study, there was no significant difference in pH between the CON and AME groups. However, the pH was in the optimal range between 6.2 and 7.2, which reflected the stability of the rumen microbial ecosystem (Calsamiglia et al., 2002). NH_3_-N is an essential indicator of rumen function and is the primary substrate for microbial protein synthesis (Wallace et al., 1994). The AME group had a lower concentration of NH_3_-N, which may be attributed to the high content of flavonoids in the AME. A similar result was found by Jelali and Ben Salem (2014) in a study with buffalo calves offered *Moringa oleifera* leaf that is rich in flavonoids. Liu et al. (2020) also reported that flavonoids decrease ruminal NH_3_-N concentrations in *in vitro* experiments. This would indicate that the AME may reduce ruminal ammonia concentrations by increasing the utilization of amino acids or promoting the synthesis of microbial proteins.

VFAs produced by ruminal microorganisms degrade nutrients, which meet 60–80% of the energy requirements of ruminants (Lane and Jesse, 1997). In the current study, there were no differences in the TVFA, acetate, butyrate, or iso-butyrate levels or acetate/propionate ratio among the treatments. In accordance with this study, Seradj et al. (2018) suggested that citrus flavonoids did not affect the concentrations of TVFAs, butyrate and isobutyrate *in vitro*. The AME did not affect the concentration of TVFAs, probably due to a significant lack of effect on ruminal pH (Gabel et al., 2002). Furthermore, we also found that supplementation with AME in the diet decreased the concentration of propionate and increased the concentration of valerate and iso-valerate. Consistent with our results, Oskoueian et al. (2013) reported that the addition of 4.5% flavonoid substrate caused a lower propionate concentration *in vitro*. Moreover, there was no difference in acetate/propionate ratio between the CON and AME groups, although the concentration of propionate was lower in the AME group than in the CON group. Ruminal BCFAs (iso-butyrate and iso-valerate) originate from the microbial deamination of branched amino acids (Apajalahti et al., 2019). A relatively high valerate content is conducive to improving the growth performance of ruminants (Bergman, 1990).

### Effects of *Allium mongolicum* Regel ethanol extract on the abundance and diversity of rumen bacteria in lambs

Previous studies have suggested that extracts from natural plants could affect the composition of rumen microorganisms in ruminants (Jiao et al., 2021; Wang et al., 2021). *Bacteroidetes* and *Firmicutes* are the predominant phyla in the ruminal microbial community, which is consistent with our results (Scharen et al., 2017). *Proteobacteria* are positively correlated with fiber intake (Faniyi et al., 2019). In the current study, we observed that the abundances of *Bacteroidetes* were lower in the AME group than in the CON group, and the abundances of *Firmicutes, Proteobacteria*, and *Cyanobacteria* were higher. Zhan et al. (2017) reported that supplementation with flavonoid extracts from alfalfa increased the ruminal abundance of *Firmicutes* in dairy cows. Jiang et al. (2021) observed that the relative abundances of *Firmicutes* and *Proteobacteria* were relatively high in yak calves offered root extracts from *Codonopsis pilosula* that contained high concentrations of flavonoids. Consequently, the flavonoids of the AME could explain the decreased abundance of *Bacteroidetes* and increased abundances of *Firmicutes* and *Proteobacteria* in the rumen of lambs.

At the genus level, *Prevotella*, *Succiniclasticum*, and *Selenomonas* are associated with propionate production (Henderson et al., 2015; Yuste et al., 2020). In the present study, adding AME to the diet significantly decreased the relative abundances of *Prevotella*, *Succiniclasticum*, and *Selenomonas*. The reduction in the abundances of *Prevotella*, *Succiniclasticum*, and *Selenomonas* resulted in a lower concentration of propionate in the rumen. The decrease in the relative abundance of *Prevotella* (within *Bacteroidetes*) could be due to the AME decreasing the relative abundance of *Bacteroidetes* at the phylum level. In addition, the abundance of *Prevotella* was negatively associated with the ruminal pH (Chiquette et al., 2012). In this study, AME increased the ruminal pH but not significantly, which may also have causes a decrease in the relative abundance of *Prevotella*. *Charistensenellaceae_R-7_group* is involved in biofilm formation (Mao et al., 2015). Studies have suggested that flavonoids can increase the relative abundance of the *Charistensenellaceae_R-7_group* (Harlow et al., 2018; Jiang et al., 2021). However, in the current study, the relative abundance of *Charistensenellaceae_R-7_group* was lower in the AME group than in the CON group, which could be due to its growth being inhibited by other active compounds of the AME.

### The relationships of volatile fatty acid levels, rumen bacteria and branched-chain fatty acid levels

The phenotypic characteristics of ruminants are determined to some extent by rumen microbiota (Scharen et al., 2018). The feed was fermented by microorganisms in the rumen to produce precursors (e.g., propionate and butyrate) of BCFA biosynthesis. Some of the precursors are formed into BCFAs in the rumen, while the others are absorbed into the blood through the rumen wall and then transported to the liver to synthesize BCFAs (Victoria and Robert, 1993). Therefore, rumen VFAs and microorganisms are key factors that directly or indirectly affect BCFA biosynthesis. In the present study, Spearman’s rank correlation coefficient analysis was conducted to analyze the relationip between VFA levels, rumen bacteria and BCFA levels related to mutton flavor. We found that MOA and MNA levels negatively correlated with iso-butyrate and iso-valerate levels. Moreover, the relative abundances of *Prevotella*, *Christensenellaceae_R-7_group*, and *Succiniclasticum* correlated positively with MOA and MNA contents, and the *Lachnospiraceae_XPB1014_group* abundance correlated negatively with MOA and MNA levels. The relative abundance of *Lachnospiraceae_XPB1014_group* correlated negatively with the EOA content. These findings reveal the relationship between the rumen bacteria and three flavor-related BCFAs in the rumen of lambs.

## Conclusion

In the present study, a diet supplemented with AME significantly decreased the concentrations of MOA and MNA in the rumen of lambs. AME supplementation also changes the rumen fermentation pattern and the composition of rumen bacteria. Moreover, this study also found that MOA and MNA were positively correlated with *Prevotella*, *Christensenellaceae_R-7_group*, and *Succiniclasticum* but negatively correlated with iso-butyrate, iso-valerate, and *Lachnospiraceae_XPB1014_group*.

## Data availability statement

The datasets presented in this study can be found in online repositories. The names of the repository/repositories and accession number(s) can be found in the article/supplementary material.

## Ethics statement

This animal study was reviewed and approved by the Animal Care and Use Committee of Inner Mongolia Agriculture University.

## Author contributions

YZ involved in investigation, formal analysis, and writing—original draft. YmZ involved in data curation. EK involved in writing—review and editing. CB involved in supervision. CA involved in project administration and funding acquisition. All authors contributed to the article and approved the submitted version.

## Footnotes

1
https://github.com/OpenGene/fastp

2
https://ccb.jhu.edu/software/FLASH/index.shtml

3
https://sourceforge.net/projects/rdp-classifier/

4
https://www.arb-silva.de/

5
https://www.mothur.org/

6
www.majorbio.com
